# Book Notices

**Published:** 1881-06-20

**Authors:** 


### *BOOK NOTICES.*

A TREATISE on the Materia Medica and Therapeutics of the Skin; by Henry G. Piffard, A. M., M. D., Professor of Dermatology, Medical Department of the City of New York, Surgeon to Charity Hospital, etc. New York, William Wood & Co. Pressley & Blakiston, 1012 Walnut street, Philadelphia. Oc., 342 p.

We regard this as one of the most valuable works in relation to the Skin that has yet been published. It contains both a synopsis of the diseases of the skin and a therapeutical description of the various drugs and agents adapted to their treatment. The author truly remarks that "A correct knowledge of the drugs that affect the skin, and the ways in which they act, naturally precedes their application. It is equally necessary to know when and how to apply them."

The formulæ in the back of the work are varied and excellent. It is indeed a book of great merit, and should be in the library of every progressive physician.

THE DISEASES OF CHILDREN—a practical and systematic work for practitioners and students; by Wm. Henry Day, author of Headaches and their Causes, Nature and Treatment; member of the Royal College of Physicians of London, Physician to the Samaritan Hospital for Women and Children. Second edition, re-written and much enlarged. Pressley & Blakiston, 1881. Atlanta, J. J. & S. P. Richards.

Seven hundred and fifty-two royal octavo pages, a work of practical merit, being the "outcome of private and hospital practice extending over a lengthened period." It is very recent, and in most cases brings to the student the latest advances in this important department. The subjects are varied; the chapters headed with full explanatory notes, and numerous valuable formulæ are found in the back part of the volume. We feel safe in recommending this as a very timely and valuable addition to our medical literature. It should be in the library of every medical student and every practitioner.

A COMPENDIUM OF MODERN PHARMACY AND DRUGGISTS' FORMULARY, Containing the recent methods of manufacturing and preparing Extracts, Tinctures, Fluid Extracts, Flavoring Extracts, Emulsions, Perfumery and Toilet articles, Wines and Liquors. Also, Physicians' Prescriptions, Liniments, Pills, Powders, Ointments, Syrups, Antidotes to Poisons, Weights and Measures, and miscellaneous information indispensible to the pharmacist. Second edition, by Walter B. Kilner, . pharmacist. Springfield, Illinois, 1881.

We have in the above a work of 686 pages, elegantly gotten up, by a pharmacist of well known ability and undoubted reliability; a compilation of approved formulæ, of variety and composition adapted to every conceivable indication, and containing, in compact and elegant form, all the most recent and approved recipes, and the method of preparing them; the different kinds of tinctures, elixirs, syrups, perfumery, cosmetics, and, indeed, every delicate and important article usually found in the stock of every respectable druggist. A work replete with suggestions and information exceedingly valuable to the physician, and one which no progressive and reputable druggist can well afford to do without.

ANTAGONISM BETWEEN MEDICINES, AND BETWEEN REMEDIES AND DISEASES: Being the Cartwright Lectures for the year 1880; by Roberts Bartholow, M. A., M. D., LL. D., Professor of Meteria Medica and General Therapeutics in the Jefferson Medical College of Philadelphia; Fellow of the College of Physicians of Philadelphia; member of the American Philosophical Society; President of the American Neurological Association; author of a Treatise on Materia Medica and Therapeutics, and a Treatise on the Practice of Medicine, etc. New York, D. Appleton & Co., 1881. J. J. & S. P. Richards, Atlanta. Price, $1.25.

This book contains 144 octavo pages. The six lectures of which it is composed have already appeared in the journals, but are here compiled in most convenient form, They are exceedingly interesting and useful to the practitioner.

HYDROPHOBIA; by Horatio R. Bigelow, M. D., member of the Medical Association and Licentiate of the Medical Society of the District of Columbia. Philadelphia, D. G. Brinton, 115 South Seventh street, 1881.

A work of 154 octavo pages, neatly gotten up and containing full and interesting information in regard to the history, pathology, treatment, etc., of this strange and terrible malady.

